# Heparan Sulfate Facilitates Binding of hIFN*γ* to Its Cell-Surface Receptor hIFNGR1

**DOI:** 10.3390/ijms23169415

**Published:** 2022-08-20

**Authors:** Elisaveta Miladinova, Elena Lilkova, Elena Krachmarova, Kristina Malinova, Peicho Petkov, Nevena Ilieva, Genoveva Nacheva, Leandar Litov

**Affiliations:** 1 Faculty of Physics, Sofia University “St. Kliment Ohridski”, 5 James Bourchier Blvd., 1164 Sofia, Bulgaria; 2 Institute of Information and Communication Technologies, Bulgarian Academy of Sciences, 2 Acad. G. Bonchev Str., 1113 Sofia, Bulgaria; 3 Institute of Molecular Biology “Roumen Tsanev”, Bulgarian Academy of Sciences, 21 Acad. G. Bonchev Str., 1113 Sofia, Bulgaria

**Keywords:** human interferon gamma, human interferon gamma receptor, heparan sulfate, molecular dynamics simulations, sodium chlorate

## Abstract

Human interferon-gamma (hIFNγ) is a crucial signaling molecule with an important role in the initialization and development of the immune response of the host. However, its aberrant activity is also associated with the progression of a multitude of autoimmune and other diseases, which determines the need for effective inhibitors of its activity. The development of such treatments requires proper understanding of the interaction of hIFNγ to its cell-surface receptor hIFNGR1. Currently, there is no comprehensive model of the mechanism of this binding process. Here, we employ molecular dynamics simulations to study on a microscopic level the process of hIFNγ–hIFNGR1 complex formation in different scenarios. We find that the two molecules alone fail to form a stable complex, but the presence of heparan-sulfate-like oligosaccharides largely facilitates the process by both demobilizing the highly flexible C-termini of the cytokine and assisting in the proper positioning of its globule between the receptor subunits. An antiproliferative-activity assay on cells depleted from cell-surface heparan sulfate (HS) sulfation together with the phosphorylation levels of the signal transducer and activator of transcription STAT1 confirms qualitatively the simulation-based multistage complex-formation model. Our results reveal the key role of HS and its proteoglycans in all processes involving hIFNγ signalling.

## 1. Introduction

Interferon-gamma (IFNγ) is a pleiotropic signaling molecule with antiviral, antibacterial, antiparasitic, antitumor and immunomodulatory activities (for review, see [1,2]). Nonetheless, its activity is associated with the pathological progression and exacerbation of numerous autoimmune [3] and neurodegenerative diseases [4,5] and some cancers [6]. In this context, the development of inhibitors of hIFNγ signalling has the potential to offer a way to manage and treat such conditions. Pursuing such an approach requires detailed and conclusive understanding of the mechanism of action of hIFNγ on target cells. To date, there is no consistent model of this mechanism and, specifically, of the cytokine binding to its cell-surface receptor.

Under physiological conditions, hIFNγ is a homodimer composed of two 143-amino-acids-long monomers in anti-parallel orientation. It is organized as an α-helical globular bundle with two long highly positively charged unstructured C-terminal tails of variable length [7,8]. The cytokine is recognized by its own species-specific receptor (hIFNGR) [8], which is found on the surface of almost all human cells [2]. It consists of two proteins–chain-1 (hIFNGR1), responsible for ligand (hIFNγ) binding ($K_{d}$ = 0.1–1 nM), and chain-2 (hIFNGR2) [9], necessary for triggering the hIFNγ signal transduction pathway, resulting in the activation of over 200 different genes [10].

**Figure 1:**
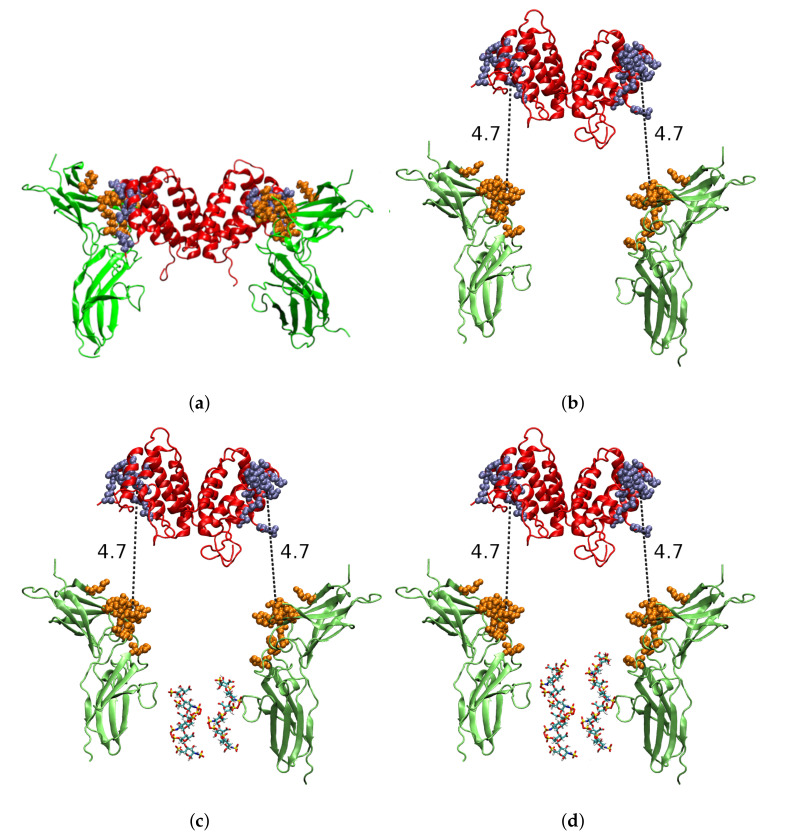
Initial models used to study the binding of hIFNγ to hIFNGR1 in different scenarios: (**a**) reference simulation based on the crystallographic structure of the complex; (**b**) binding of the full-length hIFNγ to its receptor; binding of the full-length hIFNγ to its receptor in the presence of HS-derived (**c**) hexa- or (**d**) octasaccharides. The cytokine and the receptor subunits are presented in red, resp. green cartoons. The amino acid residues in hIFNγ and hIFNGR1 molecules, participating according to [12] in the binding, are shown in blue, resp. orange spheres. The HS-derived oligosaccharides are colored by atom type and presented as licorice.

The 3D structure of the free hIFNγ homodimer [7], as well as the hIFNγ in a complex with the soluble part of the hIFNGR1 receptor [11,12] are determined by X-ray crystallography. The receptor binding sites on each side of the hIFNγ molecule are located in three distinct areas: (i) the loop between the first two N-terminal helices (residues 18–26) of one of the monomers, (ii) His${}^{111}$, and (iii) a short putative area (residues 128–131) in the flexible C-terminal domain of the other monomer (see Figure 1a for illustration). In the literature, there is controversy regarding the role of the C-termini in the cytokine–receptor binding and in the biological activity of the cytokine in general (see, e.g., [13]).

In addition to its cell-surface receptor, hIFNγ is also known to interact with high affinity with the glycosaminoglycans (GAGs) heparin (H) [14], heparan sulfate (HS) [15] and chondroitin sulfate (CS) [16]. These GAGs are linear polymers of repeating disaccharides composed of amino sugars and hexuronic acid. Each monosaccharide can be sulfated at multiple positions, which determines the high negative charge of these compounds. By attaching covalently to core proteins, GAGs form proteoglycans (PGs), which are an integral component of the basement membrane of all mammalian cells [17]. The interaction of hIFNγ and H or HS affects the cytokine’s activity dramatically, its physico-chemical properties and the proteolytic processing of its C-terminal domain [18,19,20].

Here we report our studies by means of molecular dynamics (MD) simulations and indirect in vitro experiments on the interaction of hIFNγ with hIFNGR1 in different scenarios, probing the viability of a third-party involvement in the complex-formation process. Based on our findings, we put forward the hypothesis that HSPGs act as co-receptors for the cytokine and facilitate its binding to its specific cell-surface receptor.

## 2. Results

### 2.1. Molecular Modelling

To monitor the process of cytokine–receptor binding, we used the centre-of-mass (COM) distances between the receptor-binding sites in the molecule of hIFNγ and the cytokine-binding sites in the hIFNGR1 subunits (Figure 1b), denoted $d_{1}$ and $d_{2}$. In the initial conformations of the binding simulations, these quantities had a value of 4.7 nm. In the reference simulation of the complex, $d_{1}$ had an average value of 6.87 ± 0.56 nm, and $d_{2}$ = 6.58 ± 0.24 nm.

**Figure 2:**
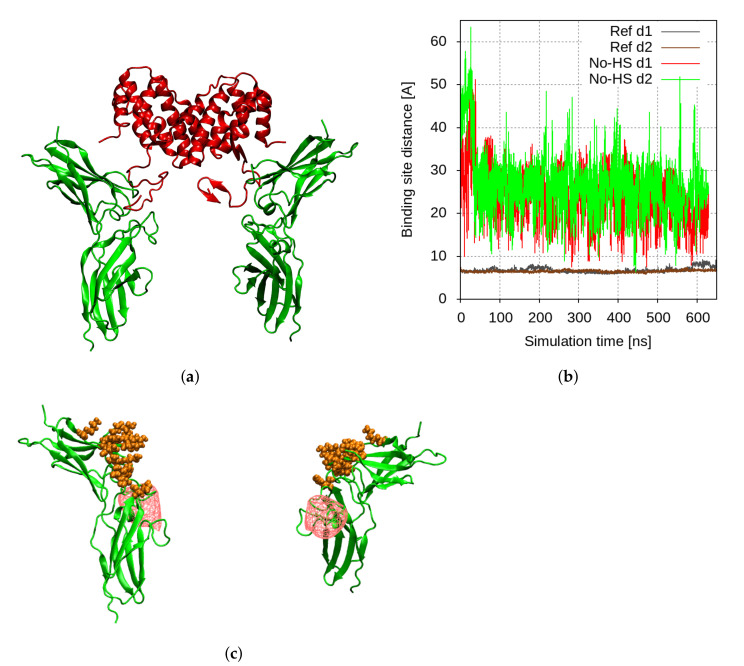
(**a**) Final conformation of the hIFNγ–hIFNGR1 binding simulation. The cytokine and the receptor subunits are presented in red, resp. green cartoons; (**b**) time evolution of the COM distances $d_{1}$ and $d_{2}$ between the binding sites in the hIFNγ and hIFNGR1 molecules with no HS-derived oligosaccharides present; (**c**) localized negative charge density at the “knees” of the two hIFNGR1 subunits, shown in red wireframe; hIFNγ-binding sites are presented in orange spheres.

**Figure 3:**
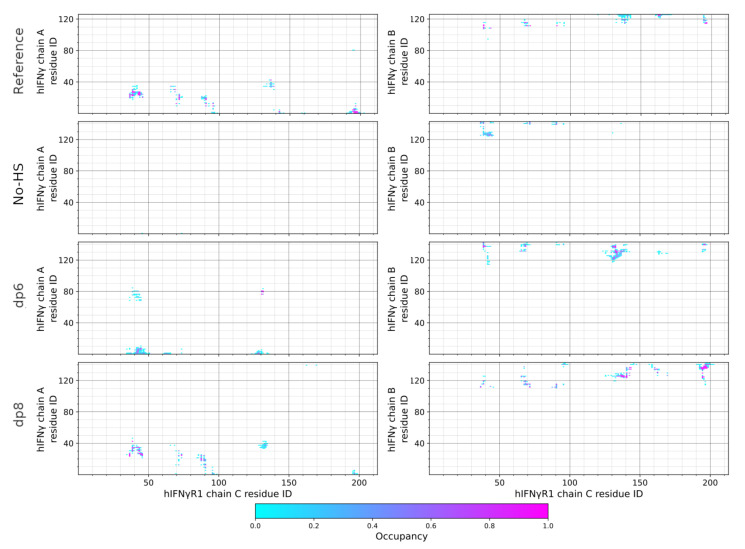
Contact maps between the two hIFNγ monomers (chain A and B) and receptor subunit C.

**Figure 4:**
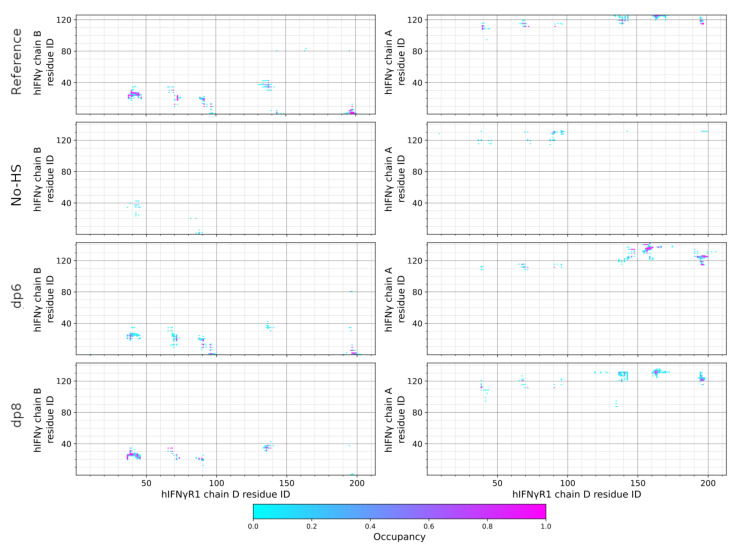
Contact maps between the two hIFNγ monomers (chain B and A) and receptor subunit D.

The interaction of the full-length hIFNγ and the two hIFNGR1 subunits does not lead to a proper formation of the complex. As seen in Figure 2a, the long unstructured and highly positively charged C-terminal tails of the two hIFNγ monomers extend downwards and move away from the globule of the cytokine to bind to two negatively charged protruding domains in the receptor subunits, which we refer to as “knees” (Figure 2c), located just below the cytokine-binding site. This keeps the globule at a distance of about 20–25 nm from the latter and prevents proper interaction of the two binding interfaces (Figure 2b). This is also reflected in the contact maps between the two hIFNγ monomers (denoted chain A and B) and the two receptor subunits (denoted chain C and D) presented in Figure 3 and Figure 4.

The contact maps show the frequency of the close contacts between the two molecules. A contact is considered present if any two heavy atoms of a hIFNγ monomer and a receptor subunit are within a cutoff radius of 4.5 Å. For this analysis, only the last 150 ns of each trajectory were employed. The contact maps were generated with the MDTraj package [21].

As seen in Figure 3, the N-terminal part of hIFNγ monomer A does not form any contacts with chain C of the receptor. Some contacts are formed between the C-terminal part of the B monomer of the cytokine and receptor subunit C, but they do not include the crucial His${}^{111}$ and its surrounding residues. As to the other binding interface, the N-terminal part of hIFNγ monomer B does form some contacts with chain D of the receptor, but these are not properly populated and are very transient in nature. This is also the case for the interaction of this receptor subunit and the C-terminal part of hIFNγ monomer A (Figure 4).

These results indicate that the interaction between hIFNγ and hIFNGR1 falls short for an efficient and proper formation of a cytokine–receptor complex. A possible reason might be that the overall negative charge of the hIFNGR1 is insufficient for effective attraction and strong binding of the hIFNγ molecule. Therefore, one might assume that additional negative charges in the vicinity of hIFNGR1 are necessary for the formation of a functional hIFNγ–hIFNGR1 complex.

Based on literature data [14,22], we identified the GAGs heparin and heparan sulfate as appropriate candidates for molecules contributing with their strong negative charges to the formation of a functional hIFNγ– hIFNGR1 complex. Moreover, HS participates in the structure of HSPGs, which are ubiquitous components of basement membranes.

**Figure 5:**
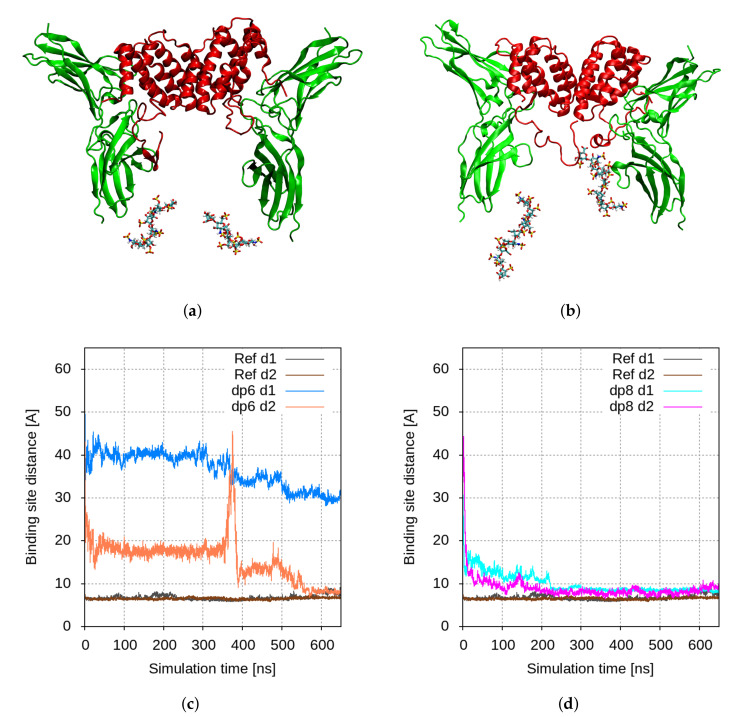
Final conformation of the hIFNγ–hIFNGR1 binding simulation in the presence of: (**a**) hexa-; or (**b**) octasaccharides. The cytokine and the receptor subunits are presented in red, resp. green cartoons, while the HS-derived oligosaccharides are colored by atom type and presented as licorice; time evolution of the COM distances $d_{1}$ and $d_{2}$ between the binding sites in the hIFNγ and hIFNGR1 molecules in the presence of: (**c**) hexa-; or (**d**) octasaccharides.

When HS-like oligosaccharides are placed between the two receptor subunits, they attract the C-termini of hIFNγ electrostatically and pull the whole molecule downward between the receptor molecules. In the dp6 case, this additional electrostatic attraction seems to be insufficient to pull the cytokine molecule strong enough; thus only one of the two binding interfaces comes to a proper contact formation. The hIFNγ globule tilts to one side so that the other binding site rotates away from the second receptor subunit (Figure 5a,c). This is also evident in Figure 3 and Figure 4 (third-row panels). While the contact maps between hIFNγ monomers and receptor subunit D resemble the reference ones (Figure 4), the interface with hIFNGR1 chain C remains largely inactive (Figure 3).

When octasaccharides are present between the receptor molecules, they manage to attract the two C-termini of the cytokine much stronger. This interaction is very intense and speedy. Within the first 100 ns, the whole globule is pulled down between the two receptor subunits (Figure 5b,d). The contact maps in the lowermost panels in Figure 3 and Figure 4 also demonstrate that in this scenario the binding interfaces adjust fairly well to each other and closely resemble the reference contact maps (top panels in Figure 3 and Figure 4).

These results suggest a tripartite model of hIFNγ–hIFNGR1 complex formation we herewith put forward: HS molecules (at least two octasaccharides per receptor unit) being located in the “bottom” of the hIFNGR1 receptor unit (corresponding to the basement cell membrane), attract the hIFNγ unstructured C-termini much stronger than the receptor itself, thus facilitating the initial stages of cytokine–receptor interaction. As a result, the hIFNγ molecule is pulled downwards to the cell surface, which favors the adoption of a correct (matching the receptor) conformation necessary for the formation of a stable and functional hIFNγ–hIFNGR1 complex.

### 2.2. The Reduction of Sulfation of HS Drastically Reduces the Levels of Phosphorylated STAT1

To investigate the effect of NaClO${}_{3}$ on the binding of hIFNγ to its cellular receptor and the activation of the hIFNγ signal transduction pathway, respectively, we monitored the level of phosphorylated STAT1 after stimulation of cells with hIFNγ. To this end, WISH cells were continuously grown in a culture medium to which NaClO${}_{3}$ was added in order to block the O-sulfation of HS [23]. Initially, the NaClO${}_{3}$ concentration was chosen based on literature data [23,24]. To ensure that the selected concentrations do not affect the cell viability, we performed an MTT assay, which is a calorimetric method for assessing the metabolic activity of cells [25]. The obtained results showed that 30 mM NaClO${}_{3}$ practically did not affect the cell viability, which allowed us to continue our experiments with this concentration (Section S2.1 and Figure S1 in the Supplementary Materials).

The formation of the hIFNγ–hIFNGR1 complex activates JAK kinases leading to phosphorylation of transcription factor STAT1 (pSTAT1), which further is translocated to the nucleus in order to induce the interferon-gamma activated genes. To investigate the level of STAT1 phosphorylation upon NaClO${}_{3}$ treatment, we performed Western blot analysis of lysates from WISH cells cultured in a medium supplemented with 30 mM NaClO${}_{3}$ and stimulated with hIFNγ (Figure S2 in the Supplementary Materials Section S2.2). The hIFNγ-stimulated cells cultured under standard conditions were used as controls. The membrane was scanned for 12 min on a C-DiGit^®^ Blot Scanner. The obtained signals were quantified using ImageJ software [26]. The signals of pSTAT1 and STAT1 for each condition were normalized to that of β-actin [27]. Then the signal of pSTAT1 was normalized to that of STAT1 since different experimental conditions may lead to a change in the expression level of STAT1. The ratio of pSTAT1 to the total amount of STAT1 for the cells cultivated in NaClO${}_{3}$ was found to be 1.7 compared to 3.14 for the control cells, which points to almost twice the reduction of the level of pSTAT1 upon NaClO${}_{3}$ treatment.

A significant decrease of the amount of pSTAT1 indicates a decrease in the formation of the hIFNγ-hIFNGR1 complex. In our experiments, this is a result of the reduction of HS sulfation after treatment of the cells with NaClO${}_{3}$, thus demonstrating the key role of HS sulfation in the cytokine–receptor complex formation.

### 2.3. Disrupting HSPG Sulfation Decreases the Antiproliferative Activity of hIFNγ

Cell-surface sulfation is mainly due to the presence of proteoglycans on the cellular membrane, in particular heparan sulfate proteoglycans. Therefore, a comparative study of the antiproliferative activity of hIFNγ in a normal cell and in cells depleted of HSPG sulfation would be indicative of the possible role HS might play in the process of cytokine–receptor binding, which is the first step in the induction of the hIFNγ signal-transduction pathway. For this purpose, we measured the antiproliferative activity of hIFNγ in treated with NaClO${}_{3}$ versus non-treated cells by a modified kynurenine bioassay [28].

**Figure 6:**
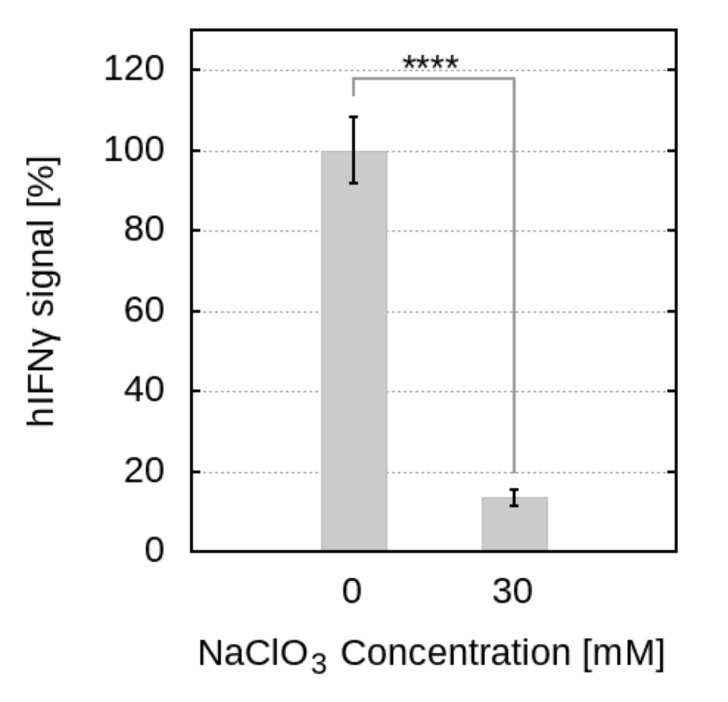
Effect of NaClO${}_{3}$ on the hIFNγ signal. After subtraction of the blank value, the absorbance obtained from cells treated with hIFNγ and cultivated in a culture medium containing 30 mM NaClO${}_{3}$ is related to that obtained from cells treated with hIFNγ only, taken as 100%. The figures shown are based on 10 independent experiments and are represented as mean ± standard error of the mean (error bars). **** *p*-value < 0.0001.

For the treated cells, cultured in a medium containing NaClO${}_{3}$, we observed a significant reduction of the hIFNγ signal, indicating an 86% lowered biological activity of the cytokine (Figure 6). The optical density at 490 nm for the untreated cells was 0.377 ± 0.031, while for the treated cells it was 0.051 ± 0.008. The difference is statistically significant with a *p*-value < 0.0001 (T = 59.99, df = 18). This observation provides further evidence for the importance of the cell surface sulfation for the interaction of the cytokine with its cellular receptor, thus supporting the MD simulation data and confirming qualitatively our hypothesis regarding the co-receptor role of HSPGs in the formation of the hIFNγ–hIFNGR1 complex.

## 3. Discussion

Note that hIFNγ is a signaling molecule, which is essential for both innate and adaptive immunity. It plays a crucial role in the modulation of the immune response against various pathogens, including viruses, bacteria and parasites. However, the activity of hIFNγ is also associated with the pathological development and progression of various autoimmune and neurodegenerative diseases, inflammation and cancer [29]. The development of therapeutic agents controlling the pathogenic effects of its aberrant actions necessitates proper understanding of how this cytokine actually binds to its extracellular receptor.

The extensive structure–functional studies do not shed much light on the biological functions of the unstructured 21 aa long C-terminal tail of the hIFNγ. One reason for this is the lack of X-ray diffraction data for this part of the molecule [7,11,12]. Three main strategies have been employed so far to study the structure–function relation of the C-terminal part of hIFNγ: (i) blocking of selected areas by sequence-specific monoclonal antibodies (MAB) [30,31]; (ii) truncation of selected regions using sequence-specific proteases [32,33]; and (iii) deletion/substitution of one or more aminoacid residues by site-directed mutagenesis [34,35]. The conclusions drawn from all these studies (in terms of significance of the unstructured C-terminal domain) vary from “extremely important” to “totally dispensable”. The hIFNγ C-terminal region is highly positively charged, which makes it highly susceptible to proteases [36]. The basic amino acids are concentrated in two domains: the first one (denoted D1) encompasses amino acids ${}^{125}$KTGKRKR${}^{131}$ and the second one (D2) includes the sequence ${}^{137}$RGRR${}^{140}$. The proteolytically sensitive segment D1 is assumed to greatly contribute to the high affinity binding of hIFNγ to the receptor and consequently to its capacity of triggering multiple cell responses [37]. Complete removal of the flexible C-terminus inactivates the cytokine. This, however, cannot explain the modulating effect of the length of the C-terminal tail on hIFNγ activity, i.e., the gradual increase in activity on removal of up to nine C-terminal amino acids (which includes D2) and the subsequent decrease in biological activity following further deletions, which renders D1 functionally more important [13].

Although numerous studies undoubtedly prove the modulating effect of the unstructured C-terminal region on hIFNγ activity, they fail to explain the molecular mechanism of its action [13]. Lortat-Jacob and collaborators were the first to realize that the role of hIFNγ C-terminus could not be explained simply by considering the hIFNγ–hIFNGR1 interaction as a protein–protein event. They published a series of papers clarifying the role of a third non-protein molecule (highly sulfated oligosaccharides) in this interaction [18,19,20,22]. In another study [38], a catch-and-release mechanism mediating a long-lived hIFNγ signalling was associated with a different negatively charged molecule, part of the cell membrane and reportedly serving as an anchor for the free cytokine molecules in the blood flow—the phosphatidylserine. The findings in [38], while supporting the idea for a third-party involvement in the signalling initiation, do not discuss the cytokine–receptor-complex formation, which is the canonical path of hIFNg signaling. It is this process and the circumstances that might facilitate or impede its flow that focus our attention in the present study.

Although hIFNγ bears four basic clusters (${}^{55}$KLFKNFK${}^{61}$, ${}^{86}$KKKR${}^{89}$, ${}^{125}$KTGKRKR${}^{131}$ and ${}^{137}$RGRR${}^{140}$) that could potentially function as HS binding sites, the specific interaction of the cytokine with HS is entirely related to the last two of them—D1 and D2 that are located within the C-terminus [15,39]. The binding constant ($K_{d}$) of hIFNγ to HS is 1.5 × 10${}^{-9}$ M [20]. The domains in HS interacting specifically with these two parts of the cytokine are highly negatively charged N-sulfated HS hexa- or octasaccharides, separated by a less charged N-acetylated-HS region [40].

Here we showed computationally that the full-length hIFNγ was unable to form a proper complex with its cell-surface receptor. The above experimental findings paved the way for the present theoretical study on the formation of the hIFNγ–hIFNGR1 complex with HS oligosaccharides being involved in this process next to the cytokine and its receptor. It should be noted that cell-surface HSPGs are known to serve as co-receptors for various signaling molecules and growth factors. HSPGs enhance their ligands’ activity by increasing their local concentration, controlling their destination and affecting their conformation, oligomerization state or stability [41].

The computer simulations described above demonstrate that the formation of a stable hIFNγ–hIFNGR1 complex is a tedious process unless an additional negatively charged molecule is present in the vicinity of the hIFNγ receptor. The minimal charge of this molecule should be comparable with the charge of the sample octasaccharide dp8 used in the simulations (−16e), and it must be located in the basement of the hIFNγ receptor unit. We hypothesize that these additional molecules are HSPGs, which are present on almost all cell membranes. The results reported here suggest that the formation of the cytokine–receptor complex is a multistage tripartite process. In the early stage, the flexible positively charged C-termini of the hIFNγ homodimer navigate the cytokine towards the receptor, being attracted by both negatively charged hIFNGR1 and HSPGs. When in close proximity to the receptor, the cytokine C-termini fall under the stronger influence of the HS electrostatic field that prevents their binding to the hIFNGR1 “knees”. At this stage, the flexible C-termini bend and pull the globular part of hIFNγ downward. When the positive charges of D1 and D2 are neutralized by HS chains, and the globular part of hIFNγ is properly situated in the cliff of the hIFNGR1 receptor, the cytokine–receptor binding interfaces are positioned close to each other, which provides for proper formation of the complex.

Our experimental data also indirectly supports this hypothesis. Inhibition of HSPG sulfation by sodium chlorate leads to reduction of hIFNγ biological activity by more than 80% and to high reduction of the level of phophorylated STAT1. We speculate that these effects are due to a hindered receptor binding because of the damaged co-receptor structure. In [42], it was experimentally confirmed that hIFNγ also interacts with another GA—chondroitin sulfate and its proteoglycans. Chondroitinase treatment of cells led to a more than 50% reduction in hIFNγ binding, and pretreatment with the enzyme significantly reduced cellular response to the cytokine. Recently, it was found that coating cells with heparin/collagen layers increases cellular response to hIFNγ, especially when the top layer was heparin [43]. These findings provide further basis for the asserted key role that HS chains play in the formation of the hIFNγ-hIFNGR1 complex.

Another cytokine, interleukin 10 (IL-10), which has a very similar structure to hIFNγ [44] and its cellular receptor (IL-10R), is in the same receptor family as hIFNGR1 and was also found to bind with high affinity to the GAGs heparin and heparan sulfate [45]. Moreover, it was demonstrated that soluble sulfated GAGs, including H, HS, CS and dermatan sulfate, inhibit IL-10 activity, similar to results on hIFNγ [46]. At the same time, inhibition of cell-surface sulfation by sodium chlorate led to a 60% decrease in the cytokine’s activity [45], which undergirds the hypothesis “that hIL-10 requires the presence of sulfated PG at the cell surface, which may in turn facilitate its interaction with high-affinity hIL-10 receptors on these cells”.

The proposed model explains the inhibitory effect of exogenous sulfated octasaccharides (such as dp8) on hIFNγ activity considered in a certain context in [47], as well as the decrease or complete loss of biological activity in constructs containing very short unstructured C-termini [35]. In the first case, the negatively charged molecules neutralize the C-terminal positive charges (i.e., they compete with the endogenous sulfated oligosaccharides fixed on the cell membrane). In the second case, the deep truncation of the C-terminus is accompanied by removal of positive charges, which are necessary for the early stages of the hIFNγ–hIFNGR1 interaction. The negative effect of the C-terminal shortening on hIFNγ biological activity is better expressed when the truncation affects the positively charged domain D1.

As mentioned above, the gradual truncation of the hIFNγ C-terminus has a two-phase effect on hIFNγ activity. We are tempted to explain the (up to 10 times) higher biological activity of hIFNγ constructs containing 6–7 aa shorter C-termini by some advantages of the shorter unstructured C-terminal tail [13]. Even after removal of 6–7 aa (including domain D2), the hIFNγ C-terminus still carries enough positive charges (mainly on account of domain D1) to recognize the hIFNGR1 receptor, to initiate hIFNγ–hIFNGR1 binding, and to decrease the probability for interaction of the C-terminus with the hIFNGR1 “knee”. The smaller size of partly truncated C-termini (probably) fits the limited space of the hIFNGR1 cliff better, thus favoring the adoption of a better hIFNγ conformation, necessary for the formation of a more stable hIFNγ–hIFNGR1 complex as compared to the full-size (143 aa) hIFNγ. We tend to disagree with Lortat-Jacob et al. [40] explaining the higher activity of truncated hIFNγ preparations with the competition of the two D1 and D2 domains for the same binding site in the hIFNGR1 subunit.

## 4. Materials and Methods

### 4.1. Molecular Dynamics Simulations

#### 4.1.1. Input Structures

The 3D structures of hIFNγ and hIFNGR1 were extracted from the Protein Data Bank [48] under PDB ID 1FG9 [12]. The 1FG9 complex was used as input for the reference simulation of the hIFNγ–hIFNGR1 complex (Figure 1a).

To study the formation of the cytokine–receptor complex, a configuration was set up in which the hIFNγ molecule was translated a few nanometers along the z-axis to distance it from the two receptors (Figure 1b). Since the 1FG9 structure does not provide information about the coordinates of the unstructured C-terminal domain, the last 18 missing aa residues were added to the protein structure, as described in [49,50]. In addition, the missing segment ${}^{141}$EVDYDP${}^{146}$ in the hIFNGR1 molecules was reconstructed using the macromolecular model building toolkit Coot [51]. The last two C-terminal amino acid residues of the two hIFNGR1 molecules were constrained to mimic reduced flexibility due to membrane attachment of the receptors.

To investigate the influence HSPGs could exert on the interaction between hIFNγ and its receptor, two models were built in which two HS-derived hexasaccharides (Figure 1c), respectively, two HS-derived octasaccharides (Figure 1d) were placed between the two receptor molecules. The 1HPN PDB entry [39] was used to develop structural models of HS-like chains with a given degree of polymerization—six (dp6), resp. eight (dp8). The Glycan Reader and Modeler module [52] of the CHARMM-GUI server [53] was used for the generation of a 3D structure, corresponding to the chosen carbohydrate sequence, as well as a topology using the latest version of the CHARMM36 carbohydrate force field [54]. The topology was converted to a GROMACS-compatible topology using the parmed module of Ambertools 16 [55]. The first monosaccharide was constrained to model immobilization of the HS chains at the cell surface.

#### 4.1.2. MD Simulation Protocol

All simulations were performed with the molecular dynamics simulation package GROMACS, version 2021.1 [56]. The protein was parameterized with the CHARMM36 protein force field [57] and the oligosaccharides with the CHARMM36 carbohydrate force field [54]. The systems were solvated in rectangular boxes with a minimal distance to the box walls of 2 nm under periodic boundary conditions. Counterions were added to all systems to neutralize their net charge. The neutralized systems were energy minimized using the steepest descent method with a maximum force tolerance of 100 kJ/(mol nm). The minimized structures were equilibrated by a short 50 ps canonical simulation at a temperature of 310 K, followed by a 200 ps isothermal-isobaric simulation at a temperature of 310 K and a pressure of 1 atm with the Berendsen thermo- and barostat [58].

For the production MD simulations, temperature and pressure were maintained by v-rescale thermostat [59] with a coupling constant of 0.25 ps and Parrinello-Rahman barostat [60] with a coupling constant of 1 ps. The leapfrog integrator [61] was used with a time-step of 2 fs, with constraints imposed on the bonds between heavy atoms and hydrogens with the help of the PLINCS algorithm [62]. Van der Waals interactions were smoothly switched off from a distance of 1.0 nm and truncated at 1.2 nm. Electrostatic interactions were treated using the smooth PME method [63] with a direct PME cut-off of 1.2 nm. Trajectory frames were recorded every 200 ps, and the simulations had a duration of 650 ns.

### 4.2. In Vitro Experiments

#### 4.2.1. Cell Culture and Phosphorylation of STAT1 after Cell Treatment with Sodium Chlorate

For the in vitro experiments, human amniotic WISH cell line (ATCC^®^ CCL-25™) was used. This cell line is used as a standard for determination of the antiviral activity of hIFNγ [64] as its epithelioid cell type [65] is a prerequisite for IFNGR enrichment, meaning high sensitivity to hIFNγ treatment. WISH cell line was propagated in Eagle’s Minimum Essential Medium (EMEM, ATCC^®^ 30-2003™) supplemented with 10% fetal bovine serum (Gibco™) and sodium chlorate (NaClO${}_{3}$, Sigma, Tokyo, Japan) to a final concentration of 30 mM. NaClO${}_{3}$ was used as a supplement throughout the whole experimental procedures in order to ensure the reduction of O-sulfation of heparan sulfate [23].

To test the cytotoxic effect of NaClO${}_{3}$ concentrations on WISH cells, MTT assay [25] was performed using Cell proliferation kit I, Roche (Section S1.1 in the Supplementary Materials). The effect of NaClO${}_{3}$ on the phosphorylation of STAT1 was studied by Western blot analysis (Section S1.2 in the Supplementary Materials).

#### 4.2.2. Biological Activity of hIFNγ after Treatment with Sodium Chlorate

WISH cells were cultured in 25 cm${}^{2}$ flasks (ThermoScientific™ Nunc™, Waltham, MA, USA) in a humidified atmosphere at 37 ${}^{{}^{\circ}}$C and 5% CO${}_{2}$, as described above. After overnight incubation, the cells were trypsinised and re-plated with a density of 1.5 × $10^{6}$ per well on a 96-well plate (Corning^®^) in a culture medium containing 30 mM NaClO${}_{3}$. On the next day, the cells were stimulated with 15 ng/mL recombinant hIFNγ, purified as described in [66]. Further, the antiproliferative activity of hIFNγ was measured by a kynurenine bioassay [67] modified as described in [28] (Section S1.3 in the Supplementary Materials).

#### 4.2.3. Statistical Analysis

The in vitro data was collected by at least three independent measurements of each data point. As presented in the Section 2, the experimental numbers and figures are based on the mean value ± the standard deviation. Statistical significance was estimated using Student’s *t*-test for independent pairs.

## 5. Conclusions

Molecular dynamics simulations were carried out to investigate the intimate mechanism of hIFNγ–hIFNGR1 interaction. As a result, a multistage tripartite model of the hIFNγ–hIFNGR1 complex formation is proposed, with heparan-sulfate proteoglycans playing a key process-promoting role as a hIFNγ co-receptor. The negatively charged sulfated oligosaccharides bind the positively charged C-termini, thus facilitating the proper positioning of the globular part of hIFNγ with respect to hIFNGR1. The experimental data presented fully supports the proposed model.

## Data Availability

Not applicable.

## Supplementary Materials

The supporting information can be downloaded at: https://www.mdpi.com/article/10.3390/ijms23169415/s1.

## Abbreviations

The following abbreviations are used in this manuscript:

aa	Amino acid
CS	Chondroitin sulfate
dp	Degree of polymerization
GAG	Glycosaminoglycans
hIFNγ	Human interferon-gamma
hIFNGR	Human interferon-gamma receptor
hIFNGR1	Human interferon-gamma receptor chain-1
hIFNGR2	Human interferon-gamma receptor chain-2
H	Heparin
HS	Heparan sulfate
HSPG	Heparan sulfate proteoglycan
IFNγ	Interferon-gamma
IL-10	Interleukine 10
MD	Molecular dynamics
PG	Proteoglycans
