# Supplementary material for: Heparan Sulfate Facilitates Binding of hIFNγ to Its Cell-Surface Receptor hIFNGR1
Source: Int J Mol Sci. 2022 Aug 20;23(16):9415. doi: 10.3390/ijms23169415 (PMC9408938; doi:10.3390/ijms23169415)
Supplement: Supplementary file 1 [file ijms-23-09415-s001.zip › ijms-1822459-supplementary.pdf]

Supplementary Materials: Heparan Sulfate Facilitates Binding of hIFNγ to its Cell-Surface Receptor hIFNGR1

Elisaveta Miladinova [1], Elena Lilkova[2,*], Elena Krachmarova[3,*], Kristina Malinova[3], Peicho Petkov[1], Nevena Ilieva[2], Genoveva Nacheva[3] and Leandar Litov [1]

S1. Materials and Methods

S1.1. MTT Test

WISH cells were seeded at a density of 5-6×10^5 cells/ml in a 6 well plate (Corning). Just before use, a sterile solution of 2.5 M $NaClO_3$ was prepared and was added to the standard media to final concentrations of 30 and 50 mM. After overnight incubation in a humidified atmosphere at 37°C and 5% CO_2, the cells were trypsinised and re-plated with the same density on a 6-well plate in a standard culture medium containing 30 and 50 mM $NaClO_3$. On the next day, the cells were trypsinised, re-plated on a 96-well plate and were serum-starved in a standard culture medium containing 30 and 50 mM $NaClO_3$ for one night. On the following day, the MMT test was performed according to the manufacturer's instructions. Briefly, 10 μl of the MTT labeling reagent (final concertation 0.5 mg/ml) were added per well and incubated for 4 hours. Then, 100 μl of the Solubilizeation solution were added to each well and incubated overnight. The formation of purple formazan crystals was measured using a microplate reader (BioTek Microplate Reader) at a wavelength of 550 nm. The reference wavelength was 630 nm. At least three independent experiments in octuplicate were performed and the statistical error was estimated.

S1.2. Western Blot Analysis

WISH cells were cultivated in a standard culture medium or in a medium containing 30 mM $NaClO_3$, as described above. After the stimulation with 15 ng/ml recombinant hIFNγ purified as described in [1], the cells were put on ice, washed with ice-cold 1 × PBS and lysed in RIPA buffer (# 9806, Cell Signaling Technology), supplemented with 1 mM PMSF (Roche), protease inhibitor cocktail (cOmplete™, Roche) and phosphatase inhibitor cocktail (Phosphatase Inhibitor Cocktail 3, Merck). After the lysis, the cells were put for 30 minutes on ice. Following the incubation, the cell lysates were centrifuged at 15 000 × g, at 4°C for 10 minutes and the supernatants were collected and denatured by addition of 5x Laemmli sample buffer. 10 μg of total cell proteins were separated via 10% SDS-PAGE and were transferred to a nitrocellulose membrane (# 12369, Cell Signaling Technology) using Mini Trans-Blot®Cell (Bio-Rad). After the transfer, the membrane was blocked for 4 hours at room temperature in blocking buffer, with composition 5% BSA in 1 × TBST (20 mM Tris, 150 mM NaCl, 0.1% w/v Tween®20). Following the blocking, the membrane was washed with 1 × TBST and incubated with primary rabbit monoclonal antibodies in the following order: pSTAT1 (Tyr 701) (#9167); STAT1 (#9175) and β-tubulin (#2146), all purchased from Cell Signaling Technology. Each incubation was performed overnight at 4°C. Anti-rabbit IgG, HRP-linked antibody (#7074, Cell Signaling Technology) was used as the secondary antibody. Since the difference in the molecular weight of pSTAT1 and STAT1 proteins is negligible, after the incubation with pSTAT1 the membrane was striped by using stripping buffer (20 mM Glycine, pH 2.5, 0.05% Tween®20 and 100 mM β-mercaptoethanol). The stripping efficiency was controlled by incubation with the secondary antibody and visualized as described below. β-tubulin level was used as a loading control. Further, the membrane was incubated for 1 minute in SignalFire™ ECL Reagent (#6883, Cell Signaling Technology) and then scanned with high sensitivity on C-DiGit®Blot Scanner. Band intensity was quantified by ImageJ software [2].

S1.3. Kynurenine Bioassay

The method is based on the ability of hIFNγ to induce the enzyme indoleamine-2,3-dioxygenase (IDO), which catalyses the oxidative degradation of the essential amino acid tryptophan to N-formylkynurenine [3], the latter leading to the so called "tryptophan starvation" and, hence, to cell death. WISH cells were cultivated in the standard culture medium. 50 μl of a cell suspension with a density of 5 $\times 10^6$ cells/ml were plated on a 96-well plate. Cells were stimulated with 15 ng/ml hIFNγ (100 μl per well). Then, 50 μl of L-tryptophan (in EMEM supplemented with 2% FBS) to final concentration of 100 μg/ml/per well was added. After incubation at humidified atmosphere at 37°C and 5% CO_2 for 72 hours, aliquots of 50 μl from each well were transferred to a new 96-well plate containing 50 μl/per well dH_2O. Then, freshly prepared Ehrlich's reagent (1.5625 g 4-dimethylaminobenzaldehyde (p-DAB, Thermo Fisher Scientific), 1.687 ml k. HCl (Sigma), 10 ml isopropanol (Sigma) and 11.687 ml dH_2O) diluted in dH_2O in a ratio of 1:1 was added. After incubation for 10 minutes, the absorbance at 490 nm was measured on ELISA reader (Bio-Tek). Unstimulated with hIFNγ cells were used as control. The mean OD_{490} values obtained by measuring the absorbance in the wells with stimulated cells were compared to the mean OD_{490} values in wells containing control cells. The experiment was performed in three independent repetitions, where each condition was repeated at least 5 times and the statistical error was estimated.

S2. Results

S2.1. Cell Viability upon Treatment with Different Concentrations of Sodium Chlorate

MTT assay was performed in order to assess the effect of $NaClO_3$ treatment on the WISH cells viability. The assay is based on the breakdown of the yellow tetrazole salt MTT to purple formazan crystals formed as a result of the metabolism of living cells. The purple formazan crystals are soluble and the resulting colored solution can be quantified using an ELISA reader. As seen in Figure S1, 30 mM $NaClO_3$ practically did not affect the cell viability, which allowed us to continue our experiments with this concentration.

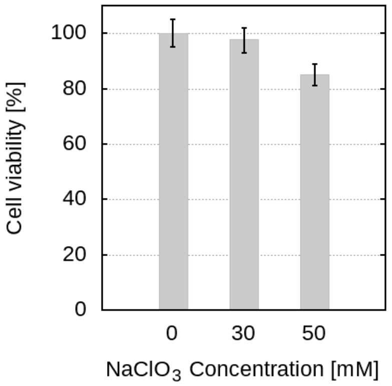

Figure S1. Survival of WISH cells cultured in medium supplemented with 30 and 50 mM $NaClO_3$. The figures shown are based on three independent experiments and are represented as mean $\pm$ standard error of the mean (error bars).

S2.2. Western Blot Analysis of the Levels of Phosphorylated STAT1 after Cell Treatment with Sodium Chlorate

To investigate the level of STAT1 phosphorylation upon $NaClO_3$ treatment we performed Western blot analysis of lysates from WISH cells cultured in a medium supplemented with 30 mM $NaClO_3$ and stimulated with hIFNγ (Fig.S2). hIFNγ-stimulated cells cultured under standard conditions were used as controls.

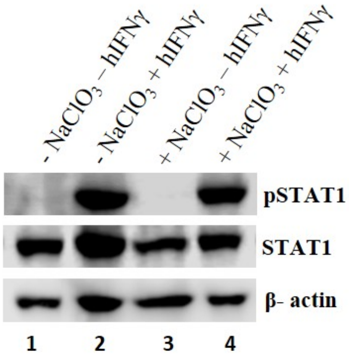

Figure S2. Western blot analysis of total cell lysates from WISH cells: 1. Non stimulated control cells cultured in standard medium; 2. Control cells cultured in standard medium and stimulated with 15 ng/ml hIFNγ; 3. Non stimulated cells cultured in a medium supplemented with 30 mM $NaClO_3$; 4. Cells cultured in a medium supplemented with 30 mM $NaClO_3$ and stimulated with 15 ng/ml hIFNγ

References

1. Tileva, M.; Krachmarova, E.; Ivanov, I.; Maskos, K.; Nacheva, G. Production of aggregation prone human interferon gamma and its mutant in highly soluble and biologically active form by SUMO fusion technology. *Protein Expr. and Purif.* **2016**, *117*, 26–34.
2. Schneider, C.; Rasband, W.; Eliceiri, K. NIH Image to ImageJ: 25 years of image analysis. *Nat. Methods* **2012**, *9*, 671–675.
3. Däubener, W.; Wanagat, N.; Pilz, K.; Seghrouchni, S.; Fischer, H.G.; Hadding, U. A new, simple, bioassay for human IFN-γ. *J. Immunol. Methods* **1994**, *168*, 39–47.
